# Widening socio-economic inequalities in oral cancer incidence in Scotland, 1976–2002

**DOI:** 10.1038/sj.bjc.6603621

**Published:** 2007-03-06

**Authors:** D I Conway, D H Brewster, P A McKinney, J Stark, A D McMahon, L M D Macpherson

**Affiliations:** 1 Dental Public Health Unit, University of Glasgow Dental School, 378 Sauchiehall Street, Glasgow G2 3JZ, UK; 2 Scottish Cancer Registry, Information Services Division, NHS National Services Scotland, Gyle Square, 1 South Gyle Crescent, Edinburgh EH12 9EB, UK; 3 Centre for Epidemiology and Biostatistics, University of Leeds, 30 Hyde Terrace, Leeds LS2 9LN, UK; 4 Robertson Centre for Biostatistics, Boyd Orr Building, University of Glasgow, University Avenue, Glasgow G12 8QQ, UK

**Keywords:** incidence, oral carcinoma, oro-pharyngeal carcinoma, Scotland, socio-economic status

## Abstract

Oral cancer incidence was investigated among 10 857 individuals using Scottish Cancer Registry data. Since 1980 the incidence of oral cancer among males in Scotland has significantly increased, the rise occurring almost entirely in the most deprived areas of residence.

Incidence of oral and oro-pharyngeal cancer continues to rise across the UK in all age groups and in both sexes (Conway *et al*, 2006). Oral cancer risk appears highly correlated with socio-economic factors, both in Scotland (Macfarlane *et al*, 1996) and in the UK (Edwards and Jones, 1999), although this was not reflected in a review of incidence studies across the world (Faggiano *et al*, 1997). Studies of the socio-economic association with oral cancer tend to be cross-sectional and cannot account for temporal changes (Greenwood *et al*, 2003; Møller and Brewster, 2005). One recent case–control study in Italy, which examined time –trends, found that oral cancer risk associated with poor socio-economic circumstances had disappeared through the 1990s (Bosetti *et al*, 2001). The aims of this study were to assess the pattern, magnitude, and time trends of socio-economic inequalities in the distribution of oral cancer in Scotland.

## MATERIALS AND METHODS

Incident cases of oral cancers (ICD-10 C00-C06) and cancers of the oro-pharynx (C09, C10, and C14) for the period 1976–2002 were obtained from the Scottish Cancer Registry, which has a high level of case ascertainment (Brewster *et al*, 1997) and accuracy of postcodes, from which the Carstairs scores were derived (see below) (Brewster *et al*, 2002). Mid-year population estimates were derived from the Annual Reports of the Registrar General for Scotland for corresponding years (General Register Office for Scotland, 1977–2003). Annual and triennial age-standardised incidence rates by sex were calculated for the period 1977–2002 by direct standardisation to the European Standard Population (Waterhouse *et al*, 1976).

This study utilises the area-based (postcode sector) Carstairs index of deprivation, which comprises four variables from the UK decennial census: social class, unemployment, overcrowding, and car ownership (Carstairs and Morris, 1991). First, 1981, 1991, and 2001 Carstairs deprivation categories were linked with cancer registration data relating to the periods of diagnosis 1976–1985, 1986–1995, and 1996–2002, respectively. Second, to validate this approach, the 1991 census-derived Carstairs deprivation categories were applied to the whole period of diagnosis 1976–2002. There were no substantial differences observed between the two methods; therefore, only data from our first method are reported here.

Poisson regression models were used to assess the magnitude of change and significance of trends in incidence. The independent effects were analysed in a fully adjusted model, and interactions were also examined. Statistical analyses were performed on SAS version 9.1 (SAS Institute Inc.).

## RESULTS

Overall incidence of oral cancer for males was 11.1 per 100 000 (95% confidence interval 10.8, 11.3) and for females was 4.1 per 100 000 (3.9, 4.2). Incidence increased significantly in both males (+87%, *P*<0.001) and females (+65%, *P*<0.001) over the period of the study. Comparing the 3-year periods at the start and end of the period, incidence in males increased from 10.1 (10.8, 11.3) to 13.3 (12.5, 14.1) per 100,000 and in females from 2.8 (2.5, 3.2) to 5.5 (5.0, 6.0) per 100 000. The median age of diagnosis was 65.1 years.

**Figure 1:**
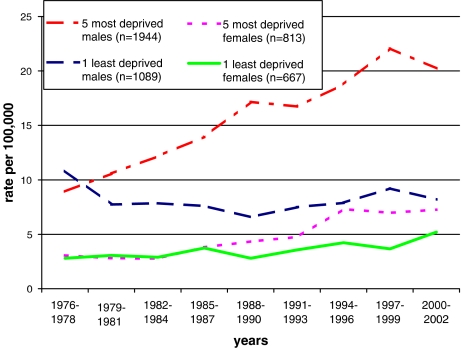
Males and females European age-standardised incidence rate (EASR) of oral and oro-pharyngeal cancer by Carstairs deprivation least and most deprived quintiles 1976–2002 in Scotland.

Between 1976 and 2002, the widening gaps in oral cancer incidence between affluent and deprived socio-economic groups by sex are clearly demonstrated in Figure 1 and Table 1. For males there was a general increase in incidence of oral cancer with increasing severity of deprivation. From an inverse relationship in 1976–1978, the gap between the most and least deprived males appeared in the late 1970s and increased rapidly through to the 1990s. The gap is almost entirely explained by an increase in incidence (+196%, *P*<0.001) in the most deprived, with a (nonsignificant) reduction in the least deprived group over the period (−74%, *P*=0.54). A different pattern is seen for females in both magnitude and timing. The incidence increased in those from all levels of deprivation, with those women from the most deprived areas having the greatest increase (+163%, *P*<0.001), but a significant increase also apparent in the least deprived (+91%, *P*<0.001). The widening gap between the most and least deprived appeared in the 1980s and continued to increase until the late 1990s. Data from 2000 to 2002 suggest that the gap had closed for women over 65 years.

## DISCUSSION

Our study shows that oral cancer incidence has increased from the 1970s to present, with corresponding widening socio-economic inequalities, particularly in males. During this period, societal changes have included post-industrial decline bringing unemployment and polarisation of poverty to the parts of Scotland (Devine and Finlay, 1996), where oral cancer is commonest.

The interaction between socio-economic life circumstances and behaviour is complex. These circumstances can affect knowledge of and ability to make healthy choices (Evans and Stoddart, 1994). Smoking (Stead *et al*, 2001) and alcohol consumption (Marmot, 1997) have been regarded as coping mechanisms for the stress associated with deprivation. It is also well documented that diet is related to access and affordability of healthy foods and not simply a lifestyle choice (Wrigley, 2002).

The important known ‘lifestyle’ factors for oral cancer are smoking (Blot *et al*, 1988) and alcohol consumption (Bagnardi *et al*, 2001), which, importantly for oral cancer, together also have a synergistic effect (Macfarlane *et al*, 1995). A diet low in fresh fruit and vegetables (Pavia *et al*, 2006) has also been found to be associated with increased oral cancer risk. The question is to what extent can the trends in oral cancer inequality be explained by socio-economic variation in these risk factors.

Data on smoking prevalence from 1972 show a consistent downward trend in all social classes in both sexes across Britain. Although smoking remained substantially lower in the higher social classes, widening inequalities were not obvious (Goddard and Green, 2005). More recent Scottish data (1995–2003) indicate that the downward trend in smoking prevalence continues with increasing inequalities in its distribution–with those from deprived areas increasingly less likely to give up (Bromley *et al*, 2005). Oral cancer risk markedly declines after quitting smoking (La Vecchia *et al*, 1999). Overall, the patterns in smoking behaviour cannot easily explain the widening socio-economic inequalities in oral cancer incidence.

The association between alcohol drinking in Scotland and socio-economic factors is somewhat mixed. The Scottish Health Survey 1995–2003 (Bromley *et al*, 2005) consistently shows that more people were drinking alcohol excessively, irrespective of their socio-economic circumstances. Similar findings in Britain from the 1980s were reported (Goddard and Green, 2005). However, between 1998 and 2003 there was a 19% rise in alcohol-related hospital episodes in Scotland with a strong relationship to area deprivation (Bromley *et al*, 2005). Thus, alcohol may have a role in socio-economic inequalities in oral cancer, although the evidence is incomplete. In our study, we were unable to investigate the well-documented synergistic effects of smoking and heavy drinking.

Daily fruit and vegetable consumption has significantly increased in Scotland (1995–2003), although those from deprived areas were eating less than their affluent counterparts (Bromley *et al*, 2005). Earlier UK data have highlighted similar trends among unemployed individuals (Braddon *et al*, 1988). However, owing to the absence of long-term dietary behaviour data, strong conclusions cannot be drawn.

The effects of socio-economic circumstances *per se* are worth considering. Although area measures of socioeconomic status have previously been criticised as ‘ecological fallacy’ – individuals are allocated a status based on their residence – this may in fact help with an explanation. People in the same area sharing many socio-economic circumstances may also experience some additional collective ‘stresses’ (Macintyre *et al*, 1993). Hypotheses of ‘biological ageing’ effects of poor socio-economic circumstances may be relevant (Adams and White, 2004), perhaps being mediated by telomere shortening (Cawthon *et al*, 2003).

Explanations for the widening inequality trends are complex. More work is required to explain the continuing rise in oral cancer incidence and the increasing socioeconomic inequalities, and public health programmes for early detection and prevention of oral cancer need to be developed by working with deprived communities.

Explanations for the widening inequality trends are complex. More work is required to explain the continuing rise in oral cancer incidence and the increasing socio-economic inequalities. Public health programmes for the early detection and prevention of oral cancer are needed in deprived communities. Although the association with socio-economic factors is well known, little has been done to explicitly address this. To date, ‘high-risk groups’ have primarily been defined by their sex, age, smoking and alcohol behaviours (Speight *et al*, 2006). Those living in deprived areas should be considered as the key ‘priority risk group’. Policy needs to be directed towards tackling root causes of disadvantage, as efforts to reduce exposure to risk factors are unlikely to succeed unless they are supported by measures designed to improve socio-economic circumstances.

## Figures and Tables

**Table 1:** Age-standardised incidence rates of oral and oro-pharyngeal cancer by sex, during nine consecutive triennia in Scotland, by Carstairs 1981 deprivation category quintile during consecutive triennia 1976–1985, Carstairs 1991 for 1986–1995 and Carstairs 2001 for 1995–2002

	**Age-standardised incidence rates per 100 000 person years at risk by period of diagnosis and ratios 5 : 1**										
**Oral cancer cases**	**1976– 1978**	**1979– 1981**	**1982– 1984**	**1985– 1987**	**1988– 1990**	**1991– 1993**	**1994– 1996**	**1997– 1999**	**2000– 2002**	**Estimated % change 1976–2002**	***P*-value for trend**
*Males* (overall)	10.1	9.3	9.6	9.8	10.7	11.2	12.2	13.8	13.3	87	<0.001
5 most deprived (*n*=1944)	8.9	10.6	12.2	14	17.2	16.7	18.8	22.1	20.2	196	<0.001
4 (*n*=1449)	9.2	8.9	9.0	9.6	11.9	12.2	11.7	14.3	13.9	83	<0.001
3 (*n*=1431)	9.1	10.8	9.6	9.4	9.5	10.6	12.8	13.3	13.4	78	<0.001
2 (*n*=1322)	11.5	8.2	9.4	8.5	7.9	9.1	10.1	10.9	11.2	23	0.05
1 least deprived (*n*=1089)	10.8	7.7	7.8	7.6	6.6	7.5	7.9	9.2	8.2	−27	0.54
Ratio 5 : 1	0.8	1.4	1.6	1.8	2.6	2.2	2.4	2.4	2.5	—	—
											
*Females* (overall)	2.8	3	3.2	3.5	3.8	4.2	5.2	5.2	5.5	65	<0.001
5 most deprived (*n*=813)	3.1	2.8	2.8	3.8	4.3	4.8	7.3	7	7.3	163	<0.001
4 (*n*=781)	2.9	3.7	3.5	3	4.2	4.1	5.8	5.6	6.5	110	<0.001
3 (*n*=660)	2.6	2.3	3.3	3.3	3.7	4	4	5	4.6	105	<0.001
2 (*n*=681)	2.2	2.8	3.3	3.4	3.7	4.4	4.4	4.5	4.1	98	<0.001
1 least deprived (*n*=667)	2.8	3.1	2.9	3.7	2.8	3.6	4.2	3.7	5.2	91	<0.001
Ratio 5 : 1	1.1	0.9	1.0	1.0	1.5	1.3	1.7	1.9	1.4	—	—
